# De novo design of Au_36_(SR)_24_ nanoclusters

**DOI:** 10.1038/s41467-020-17132-5

**Published:** 2020-07-03

**Authors:** Xu Liu, Wen Wu Xu, Xinyu Huang, Endong Wang, Xiao Cai, Yue Zhao, Jin Li, Min Xiao, Chunfeng Zhang, Yi Gao, Weiping Ding, Yan Zhu

**Affiliations:** 1 School of Chemistry and Chemical Engineering, Nanjing University, 210093 Nanjing, China; 2 School of Physical Science and Technology, Ningbo University, 315211 Ningbo, China; 3 School of Physics, Nanjing University, 210093 Nanjing, China; 4 Zhangjiang Laboratory, Shanghai Advanced Research Institute, Chinese Academy of Sciences, 201210 Shanghai, China; 5 Tsinghua University-Peking University Joint Center for Life Sciences, School of Life Sciences, Tsinghua University, 100084 Beijing, China

**Keywords:** Chemical synthesis, Theoretical chemistry, Nanoparticles

## Abstract

The discovery of atomically precise nanoclusters is generally unpredictable, and the rational synthesis of nanoclusters guided by the theoretical design is still in its infancy. Here we present a de novo design of Au_36_(SR)_24_ nanoclusters, from theoretical prediction to experimental synthesis and characterization of their physicochemical properties. The crystal structure of an Au_36_(SR)_24_ nanocluster perfectly matches the simulated structural pattern with Au_4_ tetrahedral units along a two-dimensional growth. The Au_36_(SR)_24_ nanocluster indeed differs from its structural isomer whose kernel is dissected in an Au_4_ tetrahedral manner along a one-dimensional growth. The structural isomerism in the Au_36_(SR)_24_ nanoclusters further induces distinct differences in ultrafast electron dynamics and chirality. This work will not only promote the atomically precise synthesis of nanoclusters enlightened by theoretical science, but also open up exciting opportunities for underpinning the widespread applications of structural isomers with atomic precision.

## Abstract (web summary)

The discovery of atomically precise metal nanoclusters is generally unpredictable, and there are few examples of their rational synthesis. Here, the authors report the de novo design of Au_36_(SR)_24_ nanoclusters, from theoretical prediction to experimental synthesis and characterization of physicochemical properties.

## Introduction

Thiolate-protected atomically precise gold nanoclusters referred to as Au_*n*_(SR)_*m*_ (*n* = gold atom number; *m* = ligand number) constitute a generation of gold nanoparticles and exhibit unique physical and chemical properties in optics, electronics, catalysis, chirality, and sensing^1–9^. Since the total structure of Au_102_(SR)_44_ was resolved^10^, the synthesis of one pot for one size starts to bloom and gold nanoclusters are constantly springing up^11–18^. However, despite the significant advances in Au_*n*_(SR)_*m*_ nanocluster studies, several critical issues remain still unclear, e.g. what exactly determines the structure of nanoclusters and what are real growth behaviors of nanoclusters in the solution phase? Moreover, whether the experimentalists can synthesize the specific nanoclusters based on the theoretical design is still challenging. In this work, we first identify the most potential candidate of Au_*n*_(SR)_*m*_ nanoclusters by the grand unified model (GUM) and density functional theory (DFT) calculations. Further we synthesize this nanocluster and prove its distinct property in optics and chirality. The work demonstrates the feasibility of rational synthesis of nanoclusters based on de novo design.

In correlating the conventional characterization information with the growth mechanism of nanoclusters, extensive studies have been applied to the combination of experimental and theoretical results to explore the growth of the basic structural units into smart structures^19–28^. Among these works, Au_4_ tetrahedron identified as a building block can be assembled into a series of Au_*n*_(SR)_*m*_ nanoclusters^20,21,29–33^. Especially, the solved crystal structures of a periodic series with a unified formula of Au_8*n*+4_(TBBT)_4*n*+8_ (TBBT = 4-tert-butylbenzenethiol, *n* = 3–6) nanoclusters reflect that their kernels are adopted in the connection mode of Au_4_ tetrahedron along one dimension^32^. It should be noted that Au_28_(CHT)_20_ (the isomer of Au_28_(TBBT)_20_ to some extent, cyclohexanethiol (CHT)) also contains the one-dimensional Au_14_ kernel made up of Au_4_ units. Recently, the discovery of structural isomerism in the Au_52_(SR)_32_ provided the first example that the kernel of Au_52_(SR)_32_ isomer can be arranged in a two-dimensional growth of Au_4_ tetrahedra^33^. A question naturally is whether the Au_4_ tetrahedron-based two-dimensional growth mode can be further extended to hitherto non-synthesized isomers in the family of Au_8*n*+4_(SR)_4*n*+8_.

To address this, we initiate theoretical studies to predict the structures of a series of Au_8*n*+4_(SR)_4*n*+8_ with Au_4_ tetrahedron as the building unit in a two-dimensional growth pattern. Subsequently, we identify an Au_36_(SR)_24_ packed in two-dimensional mode that has the highest stability among all the isomers, which would be an ideal candidate for experimental synthesis to fill in one of missing isomers in the family. By calculation-assisted nanoclusters discovery, a couple of isomeric Au_36_(SR)_24_ nanoclusters protected by 3,5-dimethylbenzenethiol (DMBT) are successfully synthesized and their crystal structures accurately match the predicted ones. The Au_36_(DMBT)_24_ nanocluster packed in two-dimensional mode is chiral, whereas the other isomer whose structure is identical to the previously reported Au_36_(TBBT)_24_ is achiral. The two Au_36_(DMBT)_24_ nanoclusters also exhibit dramatic differences in ultrafast dynamics.

## Results

### Theoretical prediction

**Fig. 1:**
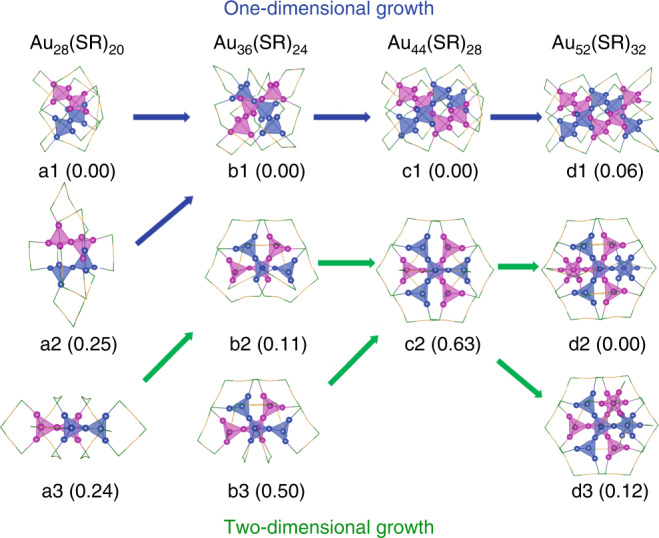
The growth patterns of Au_8*n+4*_(SR)_*4n+8*_ (*n* = 3–6) nanoclusters. The values in brackets are the relative energies (eV). Note that structures of a1/b1/c1/d1 (TBBT as ligand) and a2 (CHT as ligand) are experimentally determined. The arrows in blue and green denote the one-dimensional and two-dimensional growth, respectively. According to the relative energies, b2 of Au_36_(SR)_24_ and d2 of Au_52_(SR)_32_ are the most potential candidates among the predicted isomers. Color labels: green = S, blue/magenta/yellow = Au. The C and H atoms are omitted for clarity.

Based on GUM, there is a potential two-dimensional growth pattern, in which the growth of gold kernels in thiolate-protected gold nanoclusters can be viewed as the sequent addition of elementary blocks (tetrahedral Au_4_ in this case) obeying duet rule^21,28^. As shown in Fig. 1, there are two types of face-centered cubic (fcc) growth pattern for the kernels of Au_8*n*+4_(SR)_4*n*+8_ (*n* = 3, 4, 5, 6) using Au_4_ building blocks. Starting from the predicted Au_28_(SR)_20_ (a3 in Fig. 1)^22^, two Au_4_ units are fused with the Au_14_ kernel of Au_28_(SR)_20_ (a3 in Fig. 1) by sharing two gold atoms to form a new Au_20_ kernel, resulting in two Au_36_(SR)_24_ isomers (b2 and b3 in Fig. 1). Continuing fusing two Au_4_ units with the Au_20_ kernel of Au_36_(SR)_24_ can form an Au_44_(SR)_28_ isomer with Au_26_ kernel (c2 in Fig. 1). Following the same way, two different Au_32_ kernels can be obtained to form two Au_52_(SR)_32_ isomers (d2 and d3 in Fig. 1). Among them, the d2 of Au_52_(SR)_32_ protected by 2-phenylethanethiol (denoted as PET) was experimentally determined^33^. In this work, as illustrated in Fig. 1, totally four new isomers for Au_8*n*+4_(SR)_4*n*+8_ series have been theoretically predicted based on GUM. The series follow the two-dimensional growth mode (green arrows in Fig. 1), which is completely different from the one-dimensional double-helical growth for old series (blue arrows in Fig. 1).

All the predicted isomers (–R group is replaced by –H) were fully optimized using DFT method implemented in the Gaussian 09 program package^34^, including the TPSS functional^35^ and the all-electron basis set 6–31 G* for H and S, effective-core basis set LANL2DZ for Au. Among the predicted isomers shown in Fig. 1, the isomer b2 of Au_36_(SR)_24_ is only 0.11 eV higher in energy than b1, implying that this structure may be stable and accessible. In addition, this cluster has a large HOMO–LUMO (HOMO/LUMO: the highest/lowest occupied/unoccupied molecular orbital) gap and all positive harmonic vibrational frequencies (Supplementary Table 1), indicating its thermal and chemical stability. Thus, it motivates us to synthesize this most potential candidate based on the GUM-guided prediction.

### Synthesis and characterization

The b1 and b2 isomers in Au_36_(SR)_24_ can be simultaneously synthesized using a two-step size-focusing method when DMBT was selected as the protected ligand. The two nanoclusters were denoted as Au_36_(DMBT)_24_-1D and Au_36_(DMBT)_24_-2D according to the growth modes (vide infra). Electrospray ionization mass spectra (ESI-MS) showed the *m*/*z* 5192.02 peak with +2 charge for both nanoclusters (Supplementary Fig. 1), corresponding to [Au_36_(DMBT)_24_-2e]^2+^ (calculation: 5192.09 Da; deviation: 0.07 Da). The observed isotopic distributions were in perfect agreement with the simulated ones, further verifying the accurate formula of Au_36_(DMBT)_24_ nanoclusters.

**Fig. 2:**
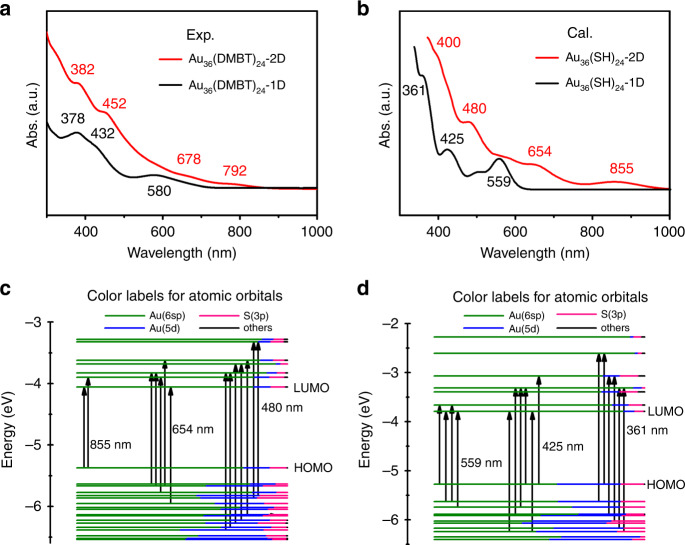
The optical absorption properties of Au_36_(DMBT)_24_ nanoclusters. **a** Experimental UV-vis spectra of Au_36_(DMBT)_24_-2D and Au_36_(DMBT)_24_-1D. **b** Calculated UV-vis spectra of Au_36_(SH)_24_-2D and Au_36_(SH)_24_-1D. The values in the black or red are the wavelength (nm) corresponding to the absorption peaks. Molecular orbital (MO) energy level diagrams for **c** Au_36_(SH)_24_-2D and **d** Au_36_(SH)_24_-1D. The energies are in eV. Various colors are used to mark relative contributions (line length with color labels) of different orbitals. The major orbital contributions from the Au(6sp), Au(5d), and S(3p) are in olive, blue, and pink, respectively. The values in the black are the wavelength (nm) corresponding to the absorption peaks.

Figure 2a showed the optical absorption spectra of Au_36_(DMBT)_24_. Au_36_(DMBT)_24_-2D exhibited the two-step peaks at 382 and 452 nm and two broad peaks at 678 and 792 nm, respectively, while Au_36_(DMBT)_24_-1D showed the two distinct peaks at 378 and 580 nm and one shoulder peak at 432 nm. The calculated UV-vis spectra of Au_36_(SH)_24_-2D and Au_36_(SH)_24_-1D were presented in Fig. 2b to reproduce the experimental results using linear response time-dependent density functional theory (TDDFT). The lowest 450 singlet-to-singlet excitation states were evaluated. As shown in Fig. 2b, the theoretical spectra can well reproduce the results of experimental measurements. Four absorption peaks (400, 480, 654, and 855 nm) for Au_36_(SH)_24_-2D and three absorption peaks (361, 425, and 559 nm) for Au_36_(SH)_24_-1D can be observed. Further examination of the Kohn–Sham (KS) molecular orbital (MO) energy levels and atomic orbital components in each KS MO of Au_36_(SH)_24_-2D and Au_36_(SH)_24_-1D indicate that the absorption peaks mainly involve the Au(*sp*) → Au(*sp*) transitions (Fig. 2c, d). Accordingly, the optical energy gaps were determined to be 1.36 eV for Au_36_(DMBT)_24_-2D and 1.71 eV for Au_36_(DMBT)_24_-1D based on the photon-energy scaled spectra (Supplementary Fig. 2), which were consistent with the corresponding calculated values of 1.31 and 1.74 eV (Supplementary Table 1).

**Fig. 3:**
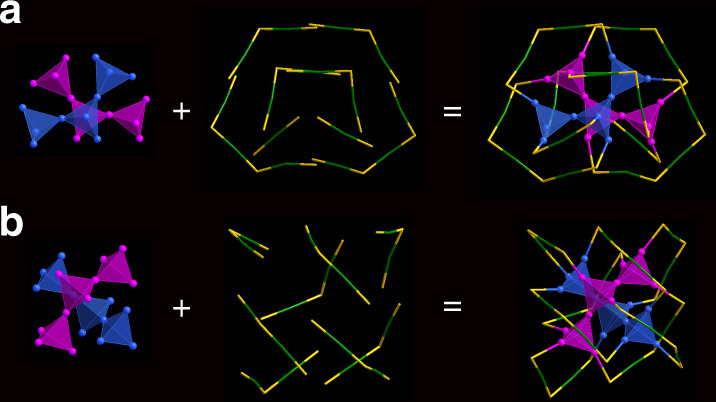
Crystal structures of the Au_36_(DMBT)_24_ nanoclusters. Structural frameworks of **a** Au_36_(DMBT)_24_-2D and **b** Au_36_(DMBT)_24_-1D shown in tetrahedral Au_4_ networks. Color labels: yellow = S, blue/magenta/green = Au. The C and H atoms are omitted for clarity.

**Fig. 4:**
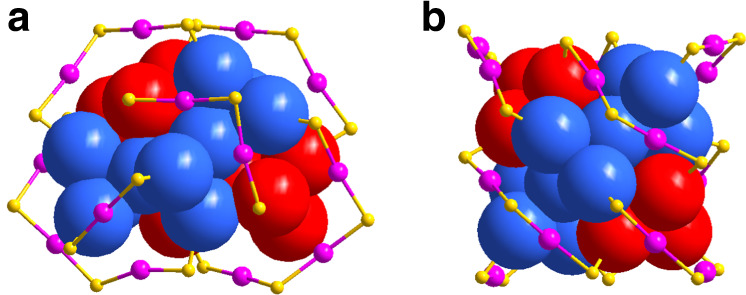
Comparison of the Au_36_(DMBT)_24_ structures. Structural frameworks of **a** Au_36_(DMBT)_24_-2D and **b** Au_36_(DMBT)_24_-1D shown in stacking mode. Color labels: yellow = S, blue/magenta/red = Au. The C and H atoms are omitted for clarity.

Single-crystal X-ray crystallography studies showed that Au_36_(DMBT)_24_-2D can be divided into a 20-atom gold kernel and eight external staple motifs (Figs. 3a and 4a). Twenty gold atoms in the kernel can be further viewed as two groups of Au_4_ tetrahedron which are arranged in a staggered mode, and this packing mode is in excellent agreement with the predicted two-dimensional growth (b2 in Fig. 1). The surface staple motifs can be divided into three categories: two monomeric Au_1_(SR)_2_, two trimeric Au_3_(SR)_4_, and four dimeric Au_2_(SR)_3_ (Figs. 3a and 4a). Note that both Au_3_(SR)_4_ and Au_2_(SR)_3_ staples are not in a plane (Supplementary Fig. 3). Alternatively, after cutting the gold atoms from Au_1_(SR)_2_ and Au_3_(SR)_4_ staples, the remaining 28 gold atoms can be constructed in a layer-by-layer fashion, which respectively consists of 6/8/8/6 atoms (Supplementary Fig. 4). This fashion is akin to a fcc structure of previously reported gold nanoclusters^11,32,33^.

As the counterpart of Au_36_(DMBT)_24_-2D, Au_36_(DMBT)_24_-1D adopts a totally different configuration from Au_36_(DMBT)_24_-2D, although it owns a 20-Au-atom kernel as well. In fact, the helical tetrahedron in the Au_36_(DMBT)_24_-1D is along the one-dimensional growth (Figs. 3b and 4b), in contrast to Au_36_(DMBT)_24_-2D (Figs. 3a and 4a), which are excellently consistent with the growing modes of the predicted b1 and b2 of Au_36_(SR)_24_ nanoclusters (Fig. 1). Notably, the Au_36_(SR)_24_-1D and Au_36_(SR)_24_-2D can be described by superatom network (SAN) model^26^ with using the adaptive natural density partitioning (AdNDP) analysis^36^ on the Au_20_^8+^ of both Au_36_(SR)_24_, which show the 12e valence electrons of Au_36_(SR)_24_ are equally distributed on six tetrahedral Au_4_ units, respectively (Supplementary Fig. 5). Thus, the Au_20_ core of each Au_36_(SR)_24_ can be viewed as a network of six 4c-2e (4c denotes 4 centers). Another notable issue is that the structural framework of Au_36_(DMBT)_24_-1D is identical to that of reported Au_36_(TBBT)_24_^11^. There are four non-planar and four co-planar staple motifs Au_2_(SR)_3_ in Au_36_(DMBT)_24_-1D (Supplementary Fig. 3). After cutting the Au atoms from co-planar Au_2_(SR)_3_ staples, the remaining 28 Au atoms of Au_36_(DMBT)_24_-1D are packed in a fcc structure (Supplementary Figs. 6 and 7). As we know, the previous studies showed the non-fcc vs. non-fcc isomerization appeared in the Au_38_(PET)_24_ isomers and the fcc vs. non-fcc isomerization existed in the Au_42_(TBBT)_26_ isomers^15,37^. Here the fcc vs. fcc isomerization is for the first time observed in the Au_36_(DMBT)_24_ isomers. In addition, Au_36_(DMBT)_24_-2D can be irreversibly converted to Au_36_(DMBT)_24_-1D at 333 K for 2 h, which indicates that the latter is more stable than the former.

To further corroborate the mechanism of two-dimensional growth in the series of Au_8*n*+4_(SR)_4*n*+8_ nanoclusters, we tried to synthesize c2 isomer of Au_44_(SR)_28_ using various synthetic methods, e.g., one-pot reduction, size-focusing, and thermal conversion (the synthesis details are shown in Methods section). Different ligands were employed such as 3-methylbenzenethiol, 4-isopropylbenzenethiol, 2,4-dimethylbenzenethiol, etc. The experimental conditions were controlled by tuning temperature and pH in different solvents. Under the above attempts, we obtained Au_36_(SR)_24_-1D, Au_44_(SR)_28_-1D, and other unknown nanoclusters, which were indicated by UV-vis (Supplementary Fig. 8) and ESI-MS spectra (Supplementary Figs. 9 and 10). The c2 isomer of Au_44_(SR)_28_ (denoted as Au_44_(SR)_28_-2D) was not obtained, which is possibly because the Au_44_(SR)_28_-2D (0.63 eV) is notably higher in energy than Au_44_(SR)_28_-1D (0 eV) (Fig. 1). In addition, as is known, the synthesis of nanoclusters involves kinetic control in the early stage and the carbon tail of the thiol ligands often plays an important role. While we believe the Au_44_(SR)_28_-2D would be attainable, their syntheses might take an exhaustive search for the right HS-R to reach the exact conditions. Therefore, we have to leave this to future work. Notably, the d2 isomer of Au_52_(SR)_32_ can be synthesized with the assistance of acid^33^. The Au_32_ kernel of Au_52_(TBBT)_32_ (d1 isomer) adopts the one-dimensional growth of the Au_4_ units (Supplementary Fig. 11a)^32^, while the kernel of Au_52_(PET)_32_ (d2 isomer) consists of 32 gold atoms that are arranged along two-dimensional direction based on the Au_4_ tetrahedron (Supplementary Fig. 11b). Thus, the Au_52_(PET)_32_ nanocluster further supports the possibility of the two-dimensional growth for Au_4_ units of Au_8*n*+4_(SR)_4*n*+8_ proposed in this work.

### Chirality

**Fig. 5:**
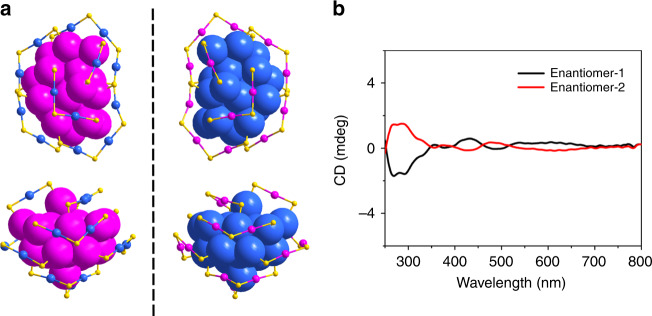
Chirality of the Au_36_(DMBT)_24_-2D nanocluster. **a** The enantiomers of Au_36_(DMBT)_24_-2D from top view (upper panel) and side view (lower panel). Color labels: yellow = S, blue/magenta = Au. The C and H atoms are omitted for clarity. **b** CD spectra of the separated enantiomers of Au_36_(DMBT)_24_-2D.

Au_36_(DMBT)_24_-2D is chiral, as the Au_20_ kernel and surface staple units are both arranged in a chiral pattern (Fig. 5a), while Au_36_(DMBT)_24_-1D is achiral due to no mirror plane occurring in the metal kernel and the surface staples (Figs. 3b and 4b). We separated the Au_36_(DMBT)_24_-2D enantiomers by high-performance liquid chromatography (HPLC) (Supplementary Fig. 12) and studied its chirality by circular dichroism (CD)^38–42^. As shown in Fig. 5b, the pair of Au_36_(DMBT)_24_-2D enantiomers showed a mirror symmetry for multiple peaks in the CD spectra, which were respectively centered at 268, 292, 329, 355, 380, 430, 487, and 615 nm in the UV-vis spectral region. The theoretically simulated CD spectra for Au_36_(SH)_24_ enantiomers agreed with the experimental spectra (Supplementary Fig. 13).

### Ultrafast electron dynamics

**Fig. 6:**
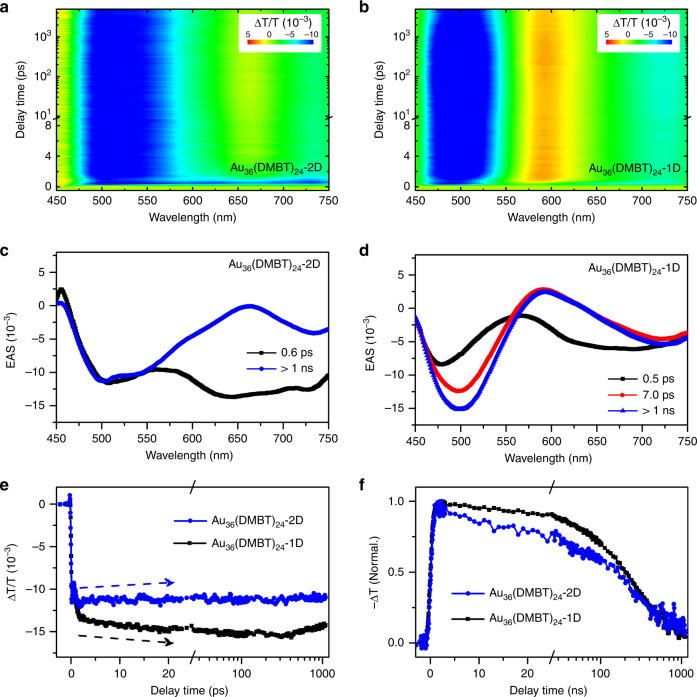
Excited-state dynamics of Au_36_(DMBT)_24_ nanoclusters. Femtosecond-resolved transient absorption data recorded from **a** Au_36_(DMBT)_24_-2D and **b** Au_36_(DMBT)_24_-1D. Spectral features of different delay components derived from global fitting analysis for **c** Au_36_(DMBT)_24_-2D and **d** Au_36_(DMBT)_24_-1D. **e** Femtosecond-resolved and **f** nanosecond-resolved dynamic traces of the two nanoclusters.

The dynamics of excited states in the two Au_36_(DMBT)_24_ nanoclusters were studied by using transient absorption (TA) spectroscopic measurements. Figure 6a, b compared the femtosecond-resolved TA data of two isomers pumped at 400 nm. We carried out the global fitting analysis to extract the evolution associated spectra (EAS) of the isomers, as shown in Fig. 6c, d. Except for some slight wavelength shifted, the spectral characteristics showed similar features for both nanoclusters with excited-state absorption near 500 nm and ground-state bleach near 600 nm, respectively. Nonetheless, the temporal dynamics exhibited remarkable differences for the two isomers. For the Au_36_(DMBT)_24_-1D nanocluster, the experimental data can be reproduced by three processes with lifetime parameters of 0.5 ps, 7 ps, and >1 ns (Fig. 6d), respectively. In analogy to the previously reported results on Au_36_(TBBT)_24_ nanocluster, the three components can be attributed to ultrafast S_n_ → S_1_ internal conversion, structural relaxation, and carrier recombination, respectively^43^. However, the component of structural relaxation (~ 7 ps) was not present in the data recorded from the Au_36_(DMBT)_24_-2D nanocluster (Fig. 6c). Such a difference was explicitly manifested in the dynamics probed at 500 nm (Fig. 6e; more meticulous comparison shown in Supplementary Fig. 14), where a delayed rise in the excited-state absorption signal observed from the Au_36_(DMBT)_24_-1D sample was absent in the data recorded from the Au_36_(DMBT)_24_-2D sample. This marked difference between the electron dynamics in the two isomers is probably related to their different structures: the picosecond component with lifetime of ~7 ps in Au_36_(DMBT)_24_-1D nanoclusters is the structural relaxation due to the expansion or torsion of one-dimensional chains after excitation^43^, while the ~7 ps component is not observed in Au_36_(DMBT)_24_-2D nanoclusters, which may be due to the limitation of the conformation changes in the two-dimensional kernel. In addition, it was found that the carrier recombination in the Au_36_(DMBT)_24_-2D sample became slightly faster than that in the Au_36_(DMBT)_24_-1D sample (Fig. 6f). These observations are interesting that Au_36_(DMBT)_24_-2D and Au_36_(DMBT)_24_-1D have same chemical compositions, but show drastic differences in their optical features and electron dynamics.

## Discussion

On the basis of the theoretical prediction for decision making, we have successfully synthesized a pair of isomeric Au_36_(DMBT)_24_ nanoclusters by selecting an appropriate ligand: one is constructed from Au_4_ tetrahedral units along the two-dimensional growth, whereas the other is patterned in an Au_4_ tetrahedral manner along the one-dimensional growth. The isomerism in the two Au_36_(DMBT)_24_ nanoclusters indeed induces evident differences in physicochemical properties, such as optics, chirality, and ultrafast dynamics. This de novo design of Au_36_(SR)_24_ is expected to stimulate further work on the synthetic chemistry guided by theoretical studies as well as on the practical applications of atomically precise isomeric nanoclusters.

## Methods

### Materials

All chemicals and reagents are commercially available and used without further purification. 3,5-dimethylbenzenethiol (3,5-DMBT, 98.0%), 4-tert-butylbenzenethiol (TBBT, 98.0%), 4-methylbenzenethiol (4-MBT, 98.0%), 3-methylbenzenethiol (3-MBT, 97.0%), 4-ethylbenzenethiol (4-EBT, 97.0%), 4-isopropylbenzenethiol (4-IPBT, 96.0%), 2,4-dimethylbenzenethiol (2,4-DMBT, 95.0%), 2-phenylethanethiol (PET, 97.0%), 4-tert-butyl benzyl mercaptan (97.0%), benzyl mercaptan (98.0%), and tetra-octylammonium bromide (TOAB, 98.0%) were purchased from Aladdin. Tetrachloroauric (III) acid (HAuCl_4_ ∙ 4H_2_O, 99.9%), sodium borohydride (NaBH_4_, 98.0%), methanol (CH_3_OH, 99.5%), dichloromethane (CH_2_Cl_2_, 99.0%), acetonitrile (CH_3_CN, 99.0%), toluene (PhCH_3_, 99.5%), and petroleum ether (AR) were obtained from Sinopharm Chemical Reagent. Co. Ltd. The water used in all experiments was ultrapure with the resistivity of 18.2 MΩ∙cm produced by a Milli-Q NANO pure water system.

### Synthesis of Au_36_(DMBT)_24_ nanoclusters

Au_36_(DMBT)_24_-2D and Au_36_(DMBT)_24_-1D nanoclusters were simultaneously synthesized using a two-step size-focusing method. Step 1: 50 mg of HAuCl_4_ ∙ 4H_2_O (0.12 mmol) dissolved in 1 mL water was mixed with 10 mL CH_2_Cl_2_ containing TOAB (80 mg, 0.15 mmol). After vigorously stirring for 20 min, the organic layer was transferred into a 50-mL flask and 60-μL 3,5-dimethylbenzenethiol (DMBT) was injected. The above mixture was stirred until the color of the solution was clear, then an aqueous solution containing 25 mg of NaBH_4_ was added at once. The reduction was allowed to proceed for 6 h. After that, the reaction mixture was dried by a rotary evaporator, and the obtained precipitates were washed with methanol three times to remove excess ligands and salts. Step 2: the obtained precursor was extracted with 0.5 mL toluene and then etched by 0.5 mL DMBT for 48 h at room temperature. The crude product was washed with CH_3_OH and separated by thin-layer chromatography. Both Au_36_(DMBT)_24_-2D and Au_36_(DMBT)_24_-1D nanoclusters were crystallized in toluene/acetonitrile solution by vapor diffusion over two weeks.

### Synthetic attempts for Au_44_(SR)_28_-2D nanoclusters

(1) Size-focusing method: the nanoclusters were prepared from the ligand (4-methylbenzenethiol, 3-methylbenzenethiol, 4-ethylbenzenethiol, 4-isopropylbenzenethiol, or 4-tert-butylphenylthiol) in place of DMBT under otherwise identical experimental conditions of Au_36_(DMBT)_24_ mentioned above. (2) One-pot reduction method: 50 mg of HAuCl_4_∙4H_2_O (0.12 mmol) and 100 mg of TOAB were dissolved in 17 mL solvent such as tetrahydrofuran, dichloromethane, or ethyl acetate. After vigorously stirring for 20 min, 190 μL organic ligand (e.g., benzyl mercaptan, 3,5-dimethylbenzenethiol, 2,4-dimethylbenzenethiol, 4-tert-butyl benzyl mercaptan, or 2-phenylethanethiol) was injected. The above mixture was stirred until the color of the solution was clear (~2 h), and then the pH was adjusted by adding H^+^ or OH^−^ prior to reduction. An aqueous solution containing 60 mg of NaBH_4_ was added in the above mixture at once and the reduction was allowed to proceed for ~14 h. After that, the solution was evaporated and the residue was washed with CH_3_OH for three times. Finally, the product was separated by thin-layer chromatography. (3) Thermal conversion method: e.g., 20 mg of Au_44_(DMBT)_28_-1D was dissolved in 2 mL toluene and 2 mL 2,4-dimethylbenzenethiol. The mixture was maintained at 60 °C for 24 h. The crude product was washed with CH_3_OH and separated by thin-layer chromatography.

### X-ray crystallography

The single-crystal X-ray diffraction data for Au_36_(DMBT)_24_-2D and Au_36_(DMBT)_24_-1D nanoclusters were collected on a Bruker D8 VENTURE using Mo Kα radiation (*λ* = 0.71073 Å) and Ga Kα radiation (*λ* = 1.34139 Å), respectively. The structure was solved by ShelxT and refined by ShelxL. Platon-Squeeze program was used to remove the contributions of disordered solvent.

### Chirality measurements

The enantiomers of Au_36_(DMBT)_24_-2D were separated by HPLC on a DIONEX UltiMate 3000 system (Thermo SCIENTIFIC) equipped with Chiral AS column (DAICEL), where the mobile phase was isopropanol/hexane = 2/98, the flow rate was set at 0.5 mL min^−1^ and the eluents were collected at different times. CD spectra were carried out on CD JASCO J-810 and the enantiomers were dissolved in dichloromethane for CD measurements.

### Ultrafast optical measurements

A Ti:sapphire regenerative amplifier (Libra, Coherent Inc.) was used for TA spectroscopy. For femtosecond TA experiment, the optical delay between the pump and probe beams was enabled by a translation stage. The pump beam at 400 nm wavelength was from the second harmonic generation of BBO crystal pumped by a portion of laser from the regenerative amplifier. The probe beam was a broadband supercontinuum light source generated by focusing a small portion of the femtosecond laser beam onto a 3-mm-thick sapphire plate. The time resolution in our fs experiments was ~150 fs. The TA signal is then analyzed by a silicon charge-coupled device (CCD; S11071-1104, Hamamatsu) with a monochromator (Acton 2358, Princeton Instrument) at 1 kHz enabled by a custom-built control board from Entwicklungsbuero Stresing. The signal-to-noise ratio in differential transmission was better than 5 × 10^−5^ after accumulating and averaging 1000 pump-on and pump-off shots for each data point. The angle between the polarized pump and probe beams was set at the magic angle. For nanosecond TA spectroscopy, we used a sub-nanosecond laser (Picolo AOT MOPA, InnoLas) at 355 nm (pulse duration ~0.8 ns) to excite the samples. The laser was synchronized to the probe pulse with a desired delay by an electronic delay generator (SRS DG645, Stanford Research System). The diameters of the pump beam spots in fs and ns experiments were ~1 mm and 0.3 mm, respectively. The diameter of probe beam was ~0.2 mm. The pump fluence was kept ~100 μJ cm^−2^ in both experiments. The clusters dissolved in toluene were placed in 1 mm path length cuvettes for TA measurements. The optical density of samples at 550 nm was ~0.3.

## Supplementary information

Supplementary Information

## Data Availability

The X-ray crystallographic coordinates for structures reported in this study have been deposited at the Cambridge Crystallographic Data Centre (CCDC), under deposition numbers CCDC 1922740 and CCDC 1922741. These data can be obtained free of charge from The Cambridge Crystallographic Data Centre via www.ccdc.cam.ac.uk/data_request/cif.

## References

[1] Higaki T (2019). Atomically tailored gold nanoclusters for catalytic applications. Angew. Chem. Int. Ed..

[2] Kang X, Zhu M (2019). Tailoring the photoluminescence of atomically precise nanoclusters. Chem. Soc. Rev..

[3] Chakraborty I, Pradeep T (2017). Atomically precise clusters of noble metals: emerging link between atoms and nanoparticles. Chem. Rev..

[4] Zhu Y (2018). Enantioseparation of Au_20_(PP_3_)_4_Cl_4_ clusters with intrinsically chiral cores. Angew. Chem. Int. Ed..

[5] Luo Z (2012). From aggregation-induced emission of Au(I)-thiolate complexes to ultrabright Au(0)@Au(I)-thiolate core-shell nanoclusters. J. Am. Chem. Soc..

[6] Turner M (2008). Selective oxidation with dioxygen by gold nanoparticle catalysts derived from 55-atom clusters. Nature.

[7] Liu Y (2018). Central doping of a foreign atom into the silver cluster for catalytic conversion of CO_2_ toward C-C bond formation. Angew. Chem. Int. Ed..

[8] Narouz MR (2019). N-heterocyclic carbene-functionalized magic-number gold nanoclusters. Nat. Chem..

[9] Shi L (2017). Self-assembly of chiral gold clusters into crystalline nanocubes of exceptional optical activity. Angew. Chem. Int. Ed..

[10] Jadzinsky PD, Calero G, Ackerson CJ, Bushnell DA, Kornberg RD (2007). Structure of a thiol monolayer-protected gold nanoparticle at 1.1 A resolution. Science.

[11] Zeng C (2012). Total structure and electronic properties of the gold nanocrystal Au_36_(SR)_24_. Angew. Chem. Int. Ed..

[12] Zeng C, Chen Y, Kirschbaum K, Lambright KJ, Jin R (2016). Emergence of hierarchical structural complexities in nanoparticles and their assembly. Science.

[13] Zhang S (2018). Diphosphine-protected ultrasmall gold nanoclusters: opened icosahedral Au_13_ and hearted-shaped Au_8_ clusters. Chem. Sci..

[14] Shen H (2019). Highly robust but surface-active: N-heterocyclic carbene-stabilized Au_25_ nanocluster. Angew. Chem. Int. Ed..

[15] Tian S (2015). Structural isomerism in gold nanoparticles revealed by X-ray crystallography. Nat. Commun..

[16] Yang S (2015). A new crystal structure of Au_36_ with a Au_14_ kernel co-capped by thiolate and chloride. J. Am. Chem. Soc..

[17] Lei Z, Li J, Wan X, Zhang W, Wang Q (2018). Isolation and total structure determination of an all-alkynyl-protected gold nanocluster Au_144_. Angew. Chem. Int. Ed..

[18] Takano S, Hirai H, Muramatsu S, Tsukuda T (2018). Hydride-doped gold superatom (Au_9_H)^2+^: synthesis, structure, and transformation. J. Am. Chem. Soc..

[19] Pei Y, Zeng X (2012). Investigating the structural evolution of thiolate protected gold clusters from first-principles. Nanoscale.

[20] Liu C, Pei Y, Sun H, Ma J (2015). The nucleation and growth mechanism of thiolate-protected Au nanoclusters. J. Am. Chem. Soc..

[21] Xu W, Zhu B, Zeng X, Gao Y (2016). A grand unified model for liganded gold clusters. Nat. Commun..

[22] Xu W, Zeng X, Gao Y (2018). The structural isomerism in gold nanoclusters. Nanoscale.

[23] Natarajan G, Mathew A, Negishi Y, Whetten RL, Pradeep T (2015). A unified framework for understanding the structure and modifications of atomically precise monolayer protected gold clusters. J. Phys. Chem. C.

[24] Aikens CM (2018). Electronic and geometric structure, optical properties, and excited state behavior in atomically precise thiolate-stabilized noble metal nanoclusters. Acc. Chem. Res..

[25] Jiang D, Overbury SH, Dai S (2013). Structure of Au_15_(SR)_13_ and its implication for the origin of the nucleus in thiolated gold nanoclusters. J. Am. Chem. Soc..

[26] Cheng L, Yuan Y, Zhang X, Yang J (2013). Superatom networks in thiolate-protected gold nanoparticles. Angew. Chem. Int. Ed..

[27] Walter M (2008). A unified view of ligand-protected gold clusters as superatom complexes. Proc. Natl Acad. Sci. USA.

[28] Xu W, Zeng X, Gao Y (2018). Application of electronic counting rules for ligand-protected gold nanoclusters. Acc. Chem. Res..

[29] Gan Z (2016). Fluorescent gold nanoclusters with interlocked staples and a fully thiolate-bound kernel. Angew. Chem. Int. Ed..

[30] Zeng C (2015). Gold tetrahedra coil up: Kekule-like and double helical superstructures. Sci. Adv..

[31] Chen Y (2016). Isomerism in Au_28_(SR)_20_ nanocluster and stable structures. J. Am. Chem. Soc..

[32] Zeng C (2016). Gold quantum boxes: on the periodicities and the quantum confinement in the Au_28_, Au_36_, Au_44_, and Au_52_ magic series. J. Am. Chem. Soc..

[33] Zhuang S (2017). The fcc structure isomerization in gold nanoclusters. Nanoscale.

[34] Frisch MJ (2009). Gaussian 09.

[35] Tao J, Perdew JP, Staroverov VN, Scuseria GE (2003). Climbing the density functional ladder: nonempirical meta-generalized gradient approximation designed for molecules and solids. Phys. Rev. Lett..

[36] Zubarev DY, Boldyrev AI (2008). Developing paradigms of chemical bonding: adaptive natural density partitioning. Phys. Chem. Chem. Phys..

[37] Zhuang S (2019). Fcc versus non-fcc structural isomerism of gold nanoparticles with kernel atom packing dependent photoluminescence. Angew. Chem. Int. Ed..

[38] Knoppe S, Azoulay R, Dass A, Bürgi T (2012). In situ reaction monitoring reveals a diastereoselective ligand exchange reaction between the intrinsically chiral Au_38_(SR)_24_ and chiral thiols. J. Am. Chem. Soc..

[39] Knoppe S, Dolamic I, Dass A, Bürgi T (2012). Separation of enantiomers and CD spectra of Au_40_(SCH_2_CH_2_Ph)_24_: spectroscopic evidence for intrinsic chirality. Angew. Chem. Int. Ed..

[40] Dolamic I, Knoppe S, Dass A, Bürgi T (2012). First enantioseparation and circular dichroism spectra of Au_38_ clusters protected by achiral ligands. Nat. Commun..

[41] Knoppe S, Bürgi T (2014). Chirality in thiolate-protected gold clusters. Acc. Chem. Res..

[42] He X, Wang Y, Jiang H, Zhao L (2016). Structurally well-defined sigmoidal gold clusters: probing the correlation between metal atom arrangement and chiroptical response. J. Am. Chem. Soc..

[43] Zhou M (2017). Evolution of excited-state dynamics in periodic Au_28_, Au_36_, Au_44_, and Au_52_ nanoclusters. J. Phys. Chem. Lett..

