# Supplementary material for: De novo design of Au36(SR)24 nanoclusters
Source: Nat Commun. 2020 Jul 3;11:3349. doi: 10.1038/s41467-020-17132-5 (PMC7335185; doi:10.1038/s41467-020-17132-5)
Supplement: Supplementary file 1 — Supplementary Information [file 41467_2020_17132_MOESM1_ESM.pdf]

De novo design of $Au_{36}(SR)_{24}$ nanoclusters

Liu *et al*

Supplementary Methods

UV-vis absorption spectroscopy. UV-vis absorption spectra were carried out on a UV-1800 spectrophotometer (Shimadzu, Japan).

ESI-MS measurements. ESI-MS spectra were measured on a Waters QT of mass spectrometer with a Z-spray source. The samples were first dissolved in toluene (~ 1 mg mL^{-1}) and then directly infused into the chamber at 5 μL min^{-1}. The source temperature was fixed at 70 °C, the spray voltage was 2.20 kV, and the cone voltage was adjusted to 60 V.

Supplementary Figures

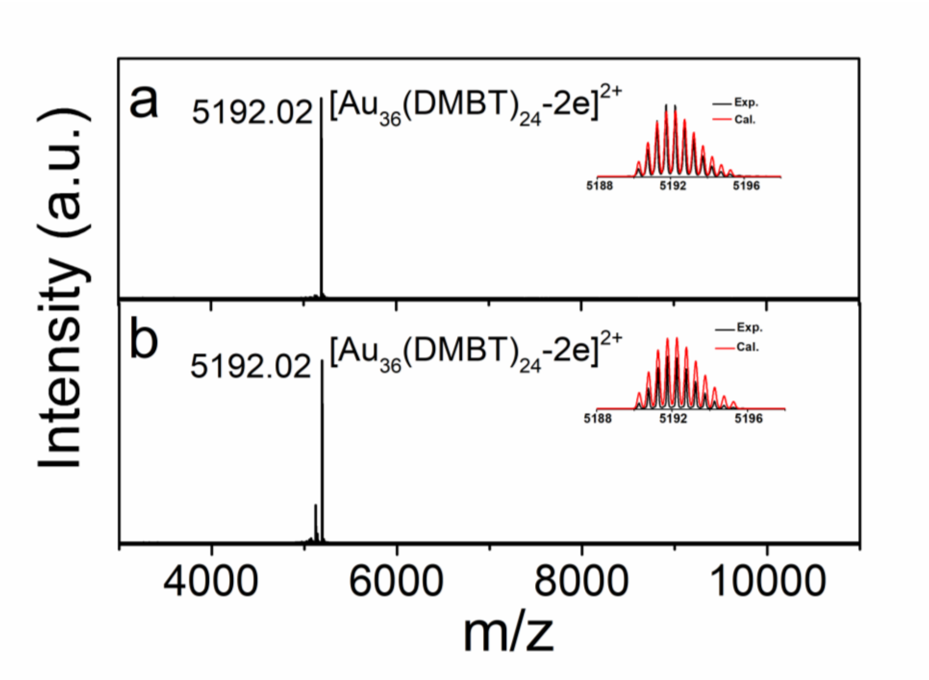

Supplementary Fig. 1 ESI-MS data of (a) $Au_{36}(DMBT)_{24}$-2D and (b) $Au_{36}(DMBT)_{24}$-1D nanoclusters.

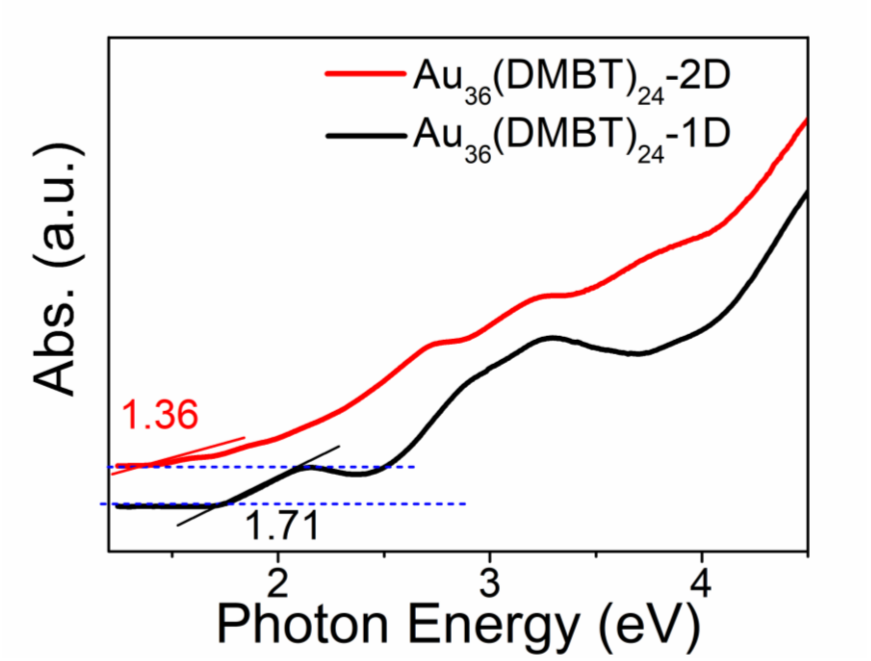

Supplementary Fig. 2 UV-vis spectra of $Au_{36}(DMBT)_{24}$-2D and $Au_{36}(DMBT)_{24}$-1D plotted on the photon energy scale.

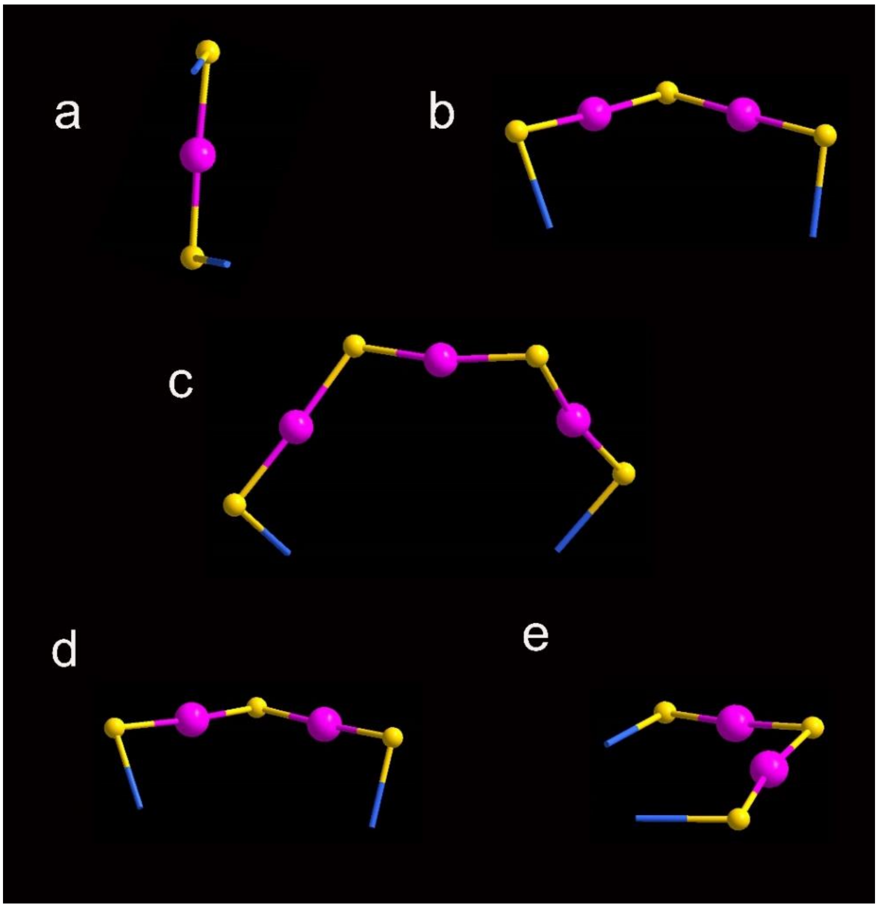

Supplementary Fig. 3 Surface staple motifs in the $Au_{36}(DMBT)_{24}$ nanoclusters: (a) monomeric $Au_1(SR)_2$ staple, (b) non-planar dimeric $Au_2(SR)_3$ staple, and (c) non-planar trimeric $Au_3(SR)_4$ staple in $Au_{36}(DMBT)_{24}$-2D; (d) non-planar dimeric $Au_2(SR)_3$ staple and (e) co-planar dimeric $Au_2(SR)_3$ staple in $Au_{36}(DMBT)_{24}$-1D. Co-planar $Au_2(SR)_3$ staples have longer Au_{staple}-Au_{kernel} distances (2.9124-4.0238 Å, averaged: 3.5632 Å) than non-planar $Au_2(SR)_3$ staples (2.8538-3.3857 Å, averaged: 3.1489 Å). Color labels: yellow = S, magenta = Au.

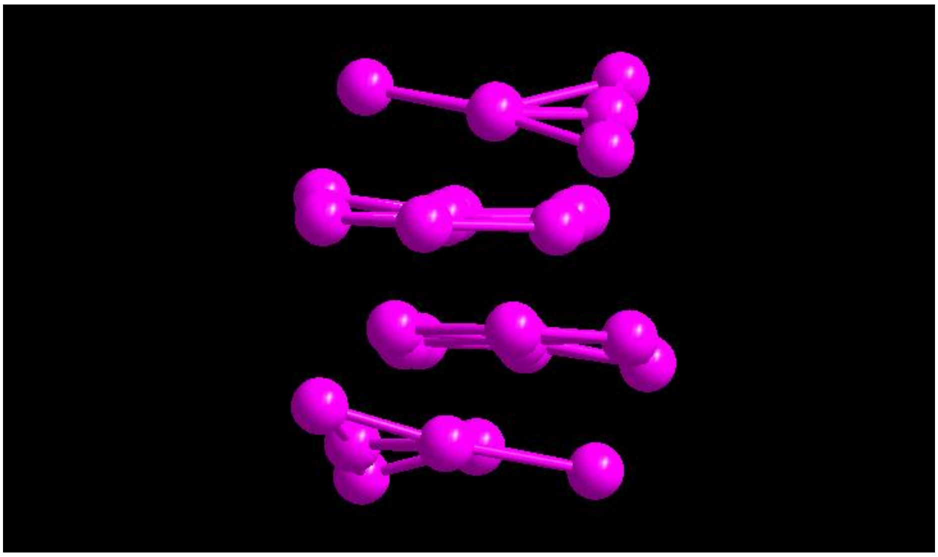

Supplementary Fig. 4 The layer-by-layer arrangement of the remaining 28 gold atoms from the $Au_{36}(DMBT)_{24}$-2D nanocluster, after cutting the Au atoms from $Au_1(SR)_2$ and $Au_3(SR)_4$ staples. Au atoms are presented in magenta.

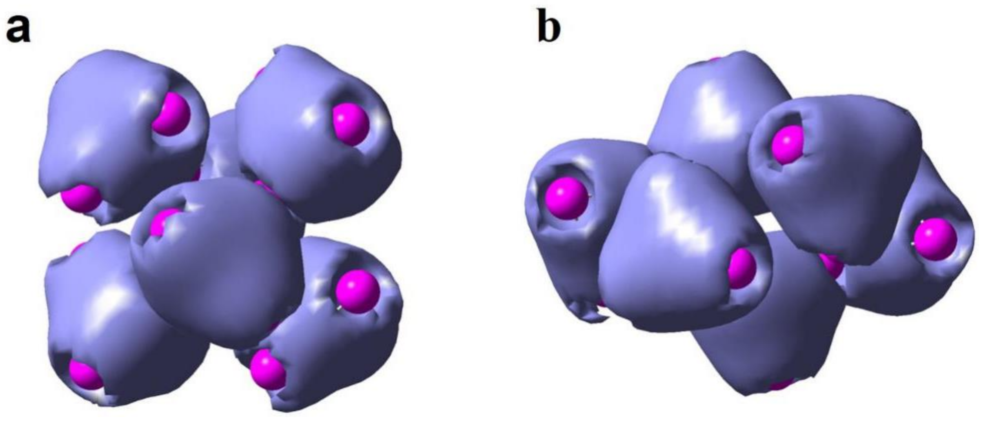

Supplementary Fig. 5 Visualization of the 12e valence electron distribution in the 6 tetrahedral Au_4 units (each unit with 2e valence electrons shown in blue) of Au_{20}^{8+} of $Au_{36}(SR)_{24}$-1D (a) and $Au_{36}(SR)_{24}$-2D (b). Au atoms are presented in wine.

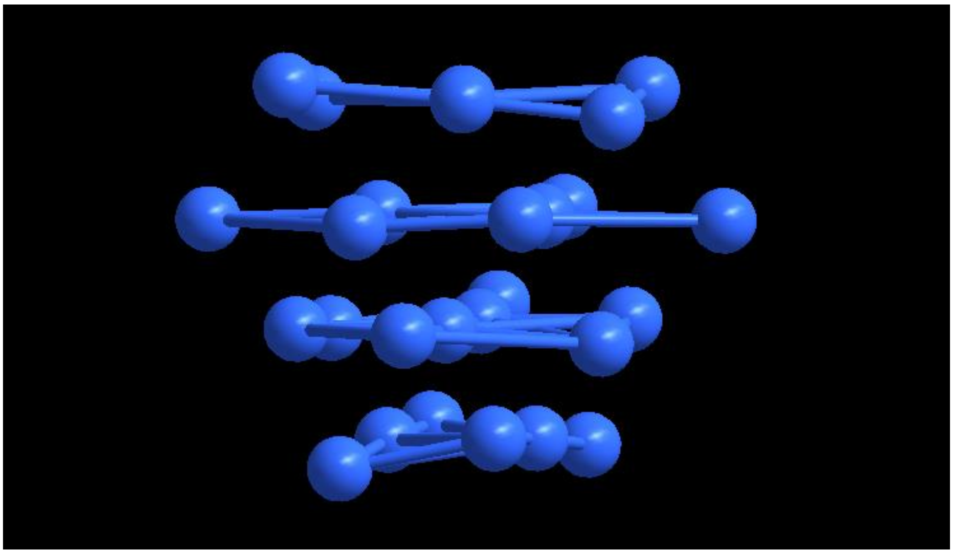

Supplementary Fig. 6 The layer-by-layer arrangement of the remaining 28 Au atoms in the $Au_{36}(DMBT)_{24}$-1D nanocluster, after cutting the Au atoms from co-planar $Au_2(SR)_3$ staples. Au atoms are presented in blue.

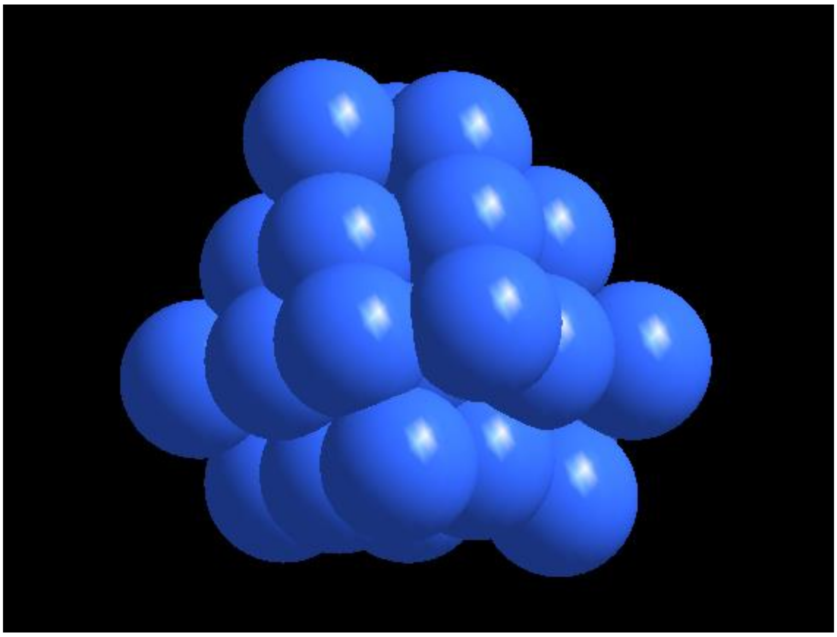

Supplementary Fig. 7 The fcc packing of the remaining 28 Au atoms in the $Au_{36}(DMBT)_{24}$-1D nanocluster, after cutting the Au atoms from co-planar $Au_2(SR)_3$ staples. Au atoms are presented in blue.

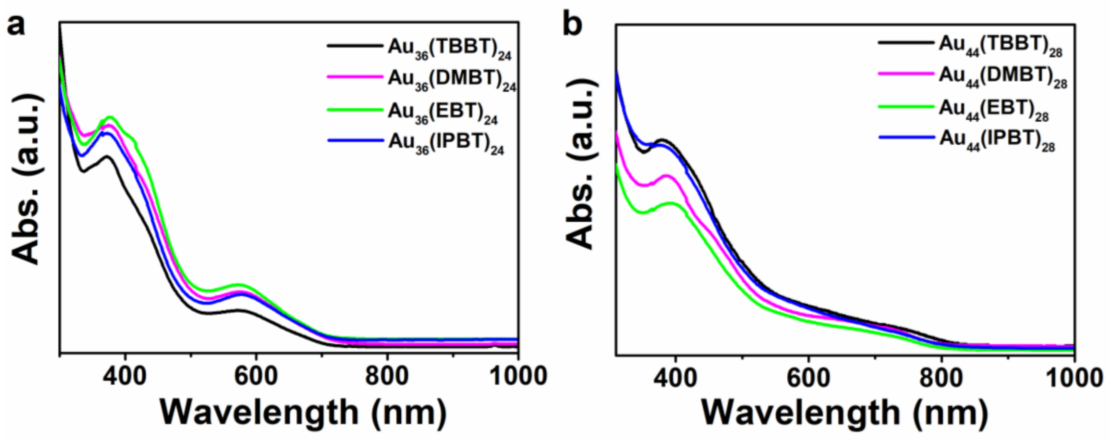

Supplementary Fig. 8 UV-vis spectra of (a) $Au_{36}(SR)_{24}$-1D and (b) $Au_{44}(SR)_{28}$-1D obtained from the synthetic attempts (size-focusing method) for $Au_{44}(SR)_{28}$-2D. TBBT = 4-tert-butylphenylthiol, DMBT = 3,5-dimethylbenzenethiol, EBT = 4-ethylbenzenethiol, and IPBT = 4-isopropylbenzenethiol.

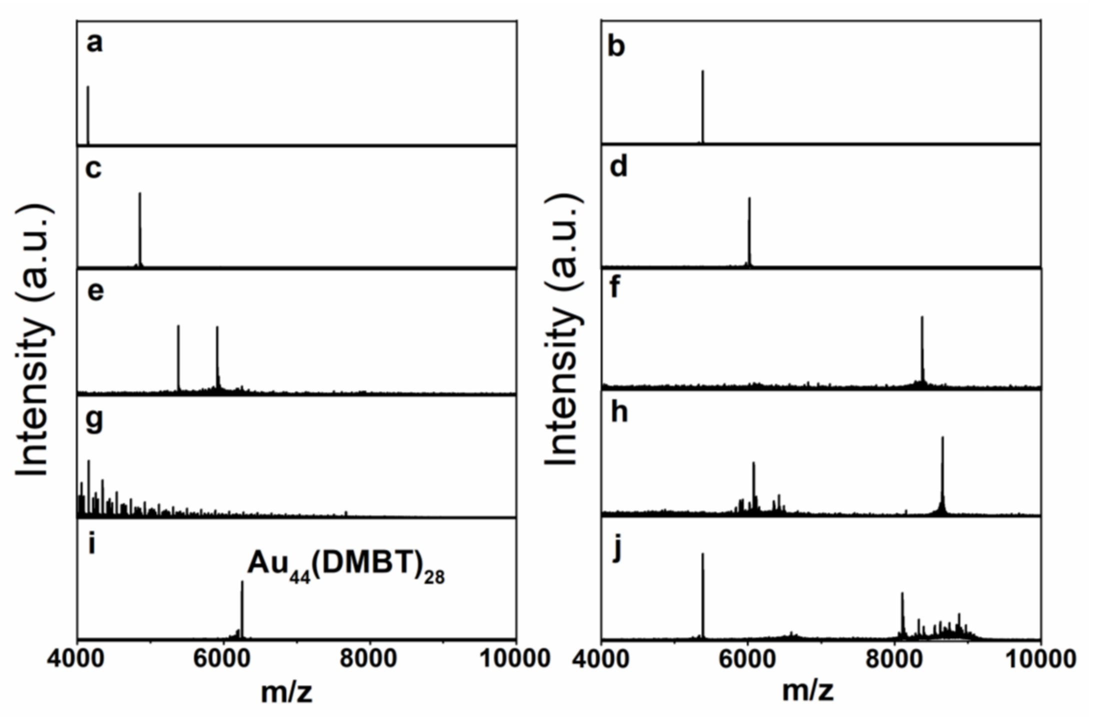

Supplementary Fig. 9 ESI-MS data of the nanoclusters obtained from the synthetic attempts for $Au_{44}(SR)_{28}$-2D. The ligands of the clusters in a and j were 2-phenylethanethiol. The ligand of the cluster in b was 2,4-dimethylbenzenethiol. The ligands of the clusters in c/d/e/f/g/i were 3,5-dimethylbenzenethiol. The ligand of the cluster in h was 4-ethylbenzenethiol. The clusters in a/b/j were synthesized via one-pot reduction method. The clusters in c/e/f/g/h/i were synthesized via size-focusing method. The cluster in d was synthesized via thermal conversion method. Note that the signal in i is assigned to $Au_{44}(DMBT)_{28}$-1D, corresponding to the pink line in Supplementary Fig. 8b.

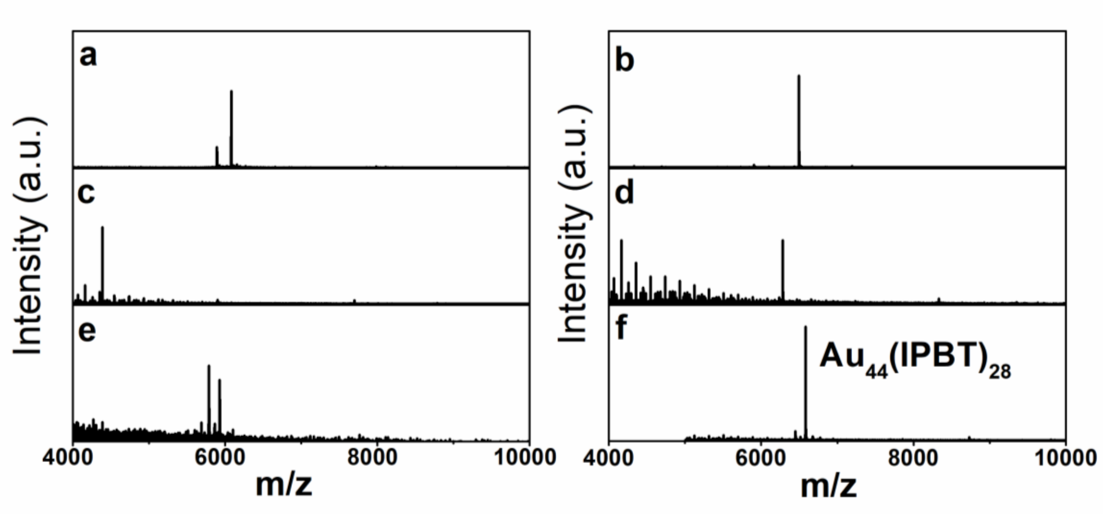

Supplementary Fig. 10 ESI-MS data of the nanoclusters obtained from the synthetic attempts for $Au_{44}(SR)_{28}$-2D. The ligand of the cluster in a was 4-tert-butyl benzyl mercaptan. The ligands of the clusters in b/c/d/e/f were 4-isopropylbenzenethiol. The cluster in a was synthesized via one-pot reduction method. The clusters in b/c/d/f were synthesized via size-focusing method. The cluster in e was synthesized via thermal conversion method. Note that the signal in f is assigned to $Au_{44}(IPBT)_{28}$-1D, corresponding to the blue line in Supplementary Fig. 8b.

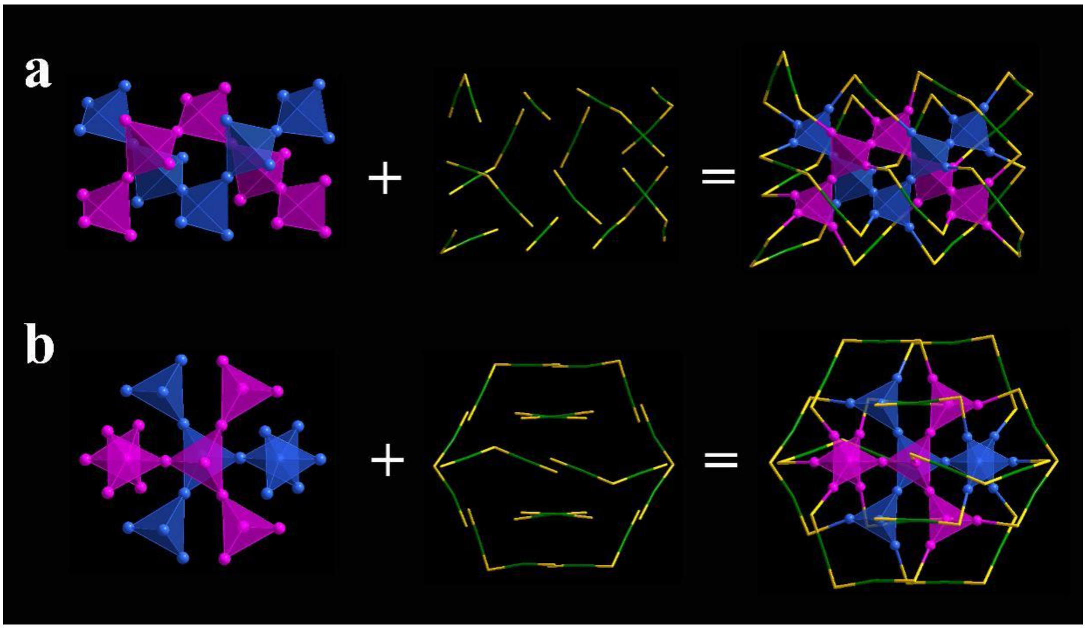

Supplementary Fig. 11 Structural frameworks of (a) $Au_{52}(TBBT)_{32}$-1D and (b) $Au_{52}(PET)_{32}$-2D shown in tetrahedral Au_4 networks. Color labels: yellow = S, blue/magenta/green = Au. The C and H atoms are omitted for clarity.

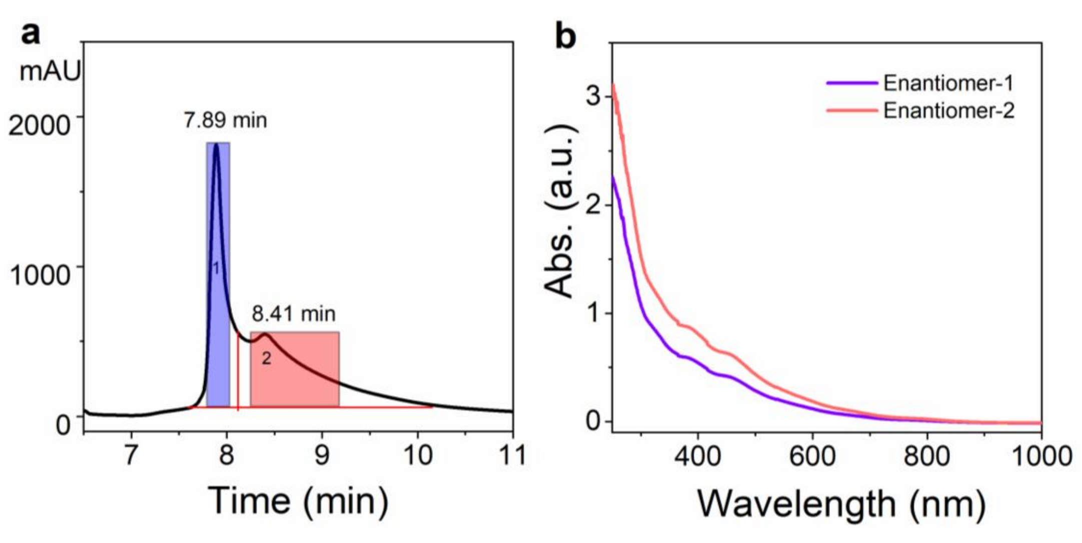

Supplementary Fig. 12 (a) The enantiomer separation of the $Au_{36}(DMBT)_{24}$-2D nanocluster by chiral-HPLC. (b) UV-vis spectra of the separated enantiomers of the $Au_{36}(DMBT)_{24}$-2D nanocluster.

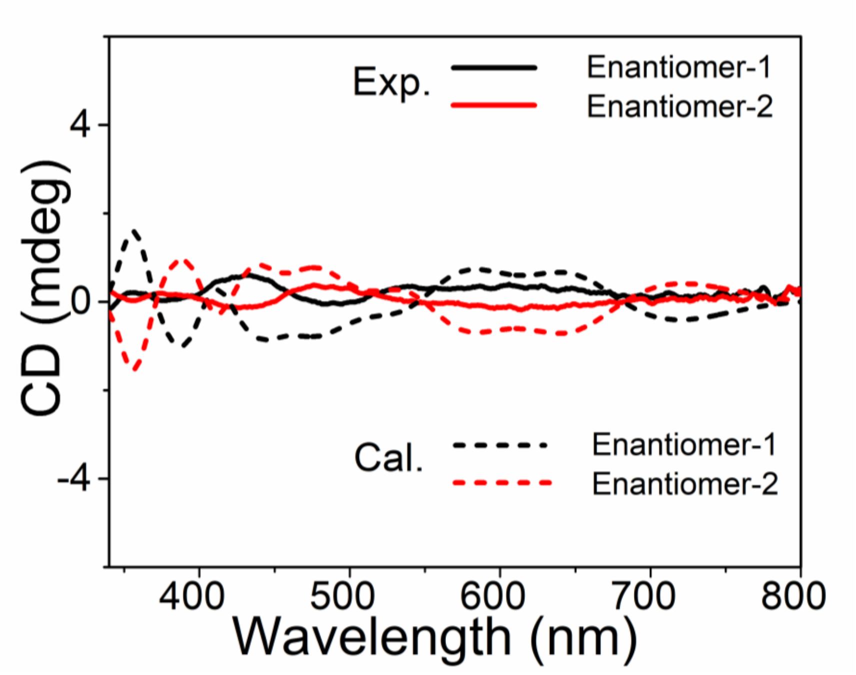

Supplementary Fig. 13 Experimental (solid lines) and calculated (dot lines) CD spectra from 340 to 800 nm of the enantiomers of $Au_{36}(DMBT)_{24}$-2D.

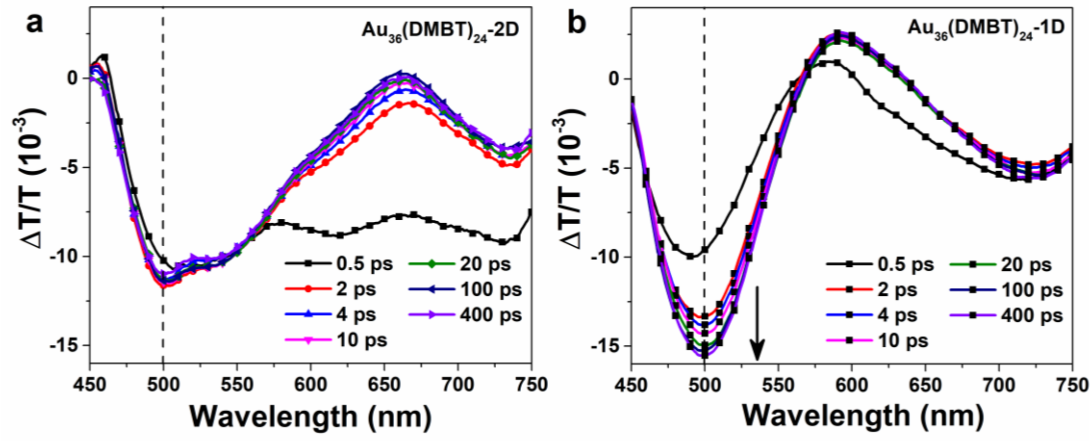

Supplementary Fig. 14 The evolution of transient absorption spectra. The transient absorption spectra of (a) $Au_{36}(DMBT)_{24}$-2D and (b) $Au_{36}(DMBT)_{24}$-1D at selected delay times. As indicated by the arrow, the spectra of $Au_{36}(DMBT)_{24}$-1D consecutively changed through 4 ps to 400 ps, especially around 500 nm, which is attributed to structual relaxation. In contrast, the spectra of $Au_{36}(DMBT)_{24}$-2D changed little from 4 ps to 400 ps.

Supplementary Tables

Supplementary Table 1. Calculated properties (relative energy in eV, HOMO/LUMO gap in eV, lowest vibrational frequency in cm^{-1}) of $Au_{8n+4}(SR)_{4n+8}$ (n = 3-6) isomers.

$Au_{8n+4}(SH)_{4n+8}$		Relative energy (eV)	HOMO/LUMO gap (eV)	Lowest vibrational frequency (cm^{-1})
$Au_{28}(SH)_{20}$	a1	0.00	1.74	8.13
	a2	0.25	1.69	8.97
	a3	0.24	1.63	4.07
$Au_{36}(SH)_{24}$	b1	0.00	1.74	7.70
	b2	0.11	1.31	12.35
	b3	0.50	1.71	5.41
$Au_{44}(SH)_{28}$	c1	0.00	1.47	7.27
	c2	0.63	1.55	11.46
$Au_{52}(SH)_{32}$	d1	0.06	1.33	7.88
	d2	0.00	1.39	9.22
	d3	0.12	1.39	9.13

Supplementary Table 2. Crystal data and structure refinement for the $Au_{36}(DMBT)_{24}$-2D nanocluster.

Empirical formula	C192 H216 Au36 S24	
Formula weight	10383.87	
Temperature	100 K	
Wavelength	0.71073 Å	
Crystal system	Triclinic	
Space group	P -1	
Unit cell dimensions	a = 17.9579(12) Å	α = 92.212(2)°
	b = 20.1056(14) Å	β = 97.095(2)°
	c = 33.879(2) Å	γ = 99.268(2)°
Volume	11958.8(14) $Å^3$	
Z	2	
Density (calculated)	2.884 g cm^{-3}	
Absorption coefficient	22.228 mm^{-1}	
F(000)	9192	
Theta range for data collection	2.025 to 24.999°	
Reflections collected	41335	
Independent reflections	41335 [R(int) = 0.0507]	
Completeness to theta = 24.999°	98.1 %	
Absorption correction	Multi-Scan	
Refinement method	Full-matrix least-squares on F^2	
Data / restraints / parameters	41335 / 9180/ 2029	
Goodness-of-fit on F^2	1.041	
Final R indices [I>2sigma(I)]	R1 = 0.0694, wR2 = 0.2096	
R indices (all data)	R1 = 0.0896, wR2 = 0.2325	
Largest diff. peak and hole	3.751 and -3.623 e$Å^{-3}$	

Supplementary Table 3. Crystal data and structure refinement for the $Au_{36}(DMBT)_{24}$-1D nanocluster.

Empirical formula	C192 H216 Au36 S24	
Formula weight	10383.87	
Temperature	253 K	
Wavelength	1.34139 Å	
Crystal system	Monoclinic	
Space group	P1 21 1	
Unit cell dimensions	a = 19.4462(10) Å	α = 90°
	b = 32.2231(15) Å	β = 90.359(2)°
	c = 19.7739(10) Å	γ = 90°
Volume	12390.4(11) $Å^3$	
Z	2	
Density (calculated)	2.783 g cm^{-3}	
Absorption coefficient	28.204 mm^{-1}	
F(000)	9192	
Theta range for data collection	1.94 to 57.02°	
Index ranges	-22<=h<=24, -34<=k<=40, -24<=l<=24	
Reflections collected	100651	
Coverage of independent reflections	99.6%	
Absorption correction	Multi-Scan	
Max. and min. transmission	0.0710 and 0.0710	
Refinement method	Full-matrix least-squares on F^2	
Data / restraints / parameters	45648 / 14564 / 2305	
Goodness-of-fit on F^2	0.948	
Final R indices [I>2sigma(I)]	R1 = 0.0485, wR2 = 0.0881	
R indices (all data)	R1 = 0.0914, wR2 = 0.1034	
Largest diff. peak and hole	1.118 and -1.485 e$Å^{-3}$	
